# Publisher Correction: Innate immune control of influenza virus interspecies adaptation via IFITM3

**DOI:** 10.1038/s41467-024-54981-w

**Published:** 2024-12-11

**Authors:** Parker J. Denz, Samuel Speaks, Adam D. Kenney, Adrian C. Eddy, Jonathan L. Papa, Jack Roettger, Sydney C. Scace, Adam Rubrum, Emily A. Hemann, Adriana Forero, Richard J. Webby, Andrew S. Bowman, Jacob S. Yount

**Affiliations:** 1 Department of Microbial Infection and Immunity, The Ohio State University College of Medicine, Columbus, OH USA; 2 Viruses and Emerging Pathogens Program, Infectious Diseases Institute, The Ohio State University, Columbus, OH USA; 3 Department of Infectious Diseases, St. Jude Children’s Research Hospital, Memphis, TN USA; 4 Department of Veterinary Preventive Medicine, The Ohio State University, Columbus, OH USA

**Keywords:** Influenza virus, Virus-host interactions

Correction to: *Nature Communications* 10.1038/s41467-024-53792-3, published online 30 October 2024

In the PDF version of this Article, Fig. 5 was cropped.

Panel labels a, c, e, and g, and the labelling of *y*-axes were missing. Additionally, Body Weight curves shown in panels b and f lacked days 8 and 6, respectively.

This has now been corrected in the PDF version of the Article.

