# Supplementary material for: Innate immune control of influenza virus interspecies adaptation via IFITM3
Source: Nat Commun. 2024 Oct 30;15:9375. doi: 10.1038/s41467-024-53792-3 (PMC11525587; doi:10.1038/s41467-024-53792-3)
Supplement: Supplementary file 1 — Supplementary Information [file 41467_2024_53792_MOESM1_ESM.pdf]

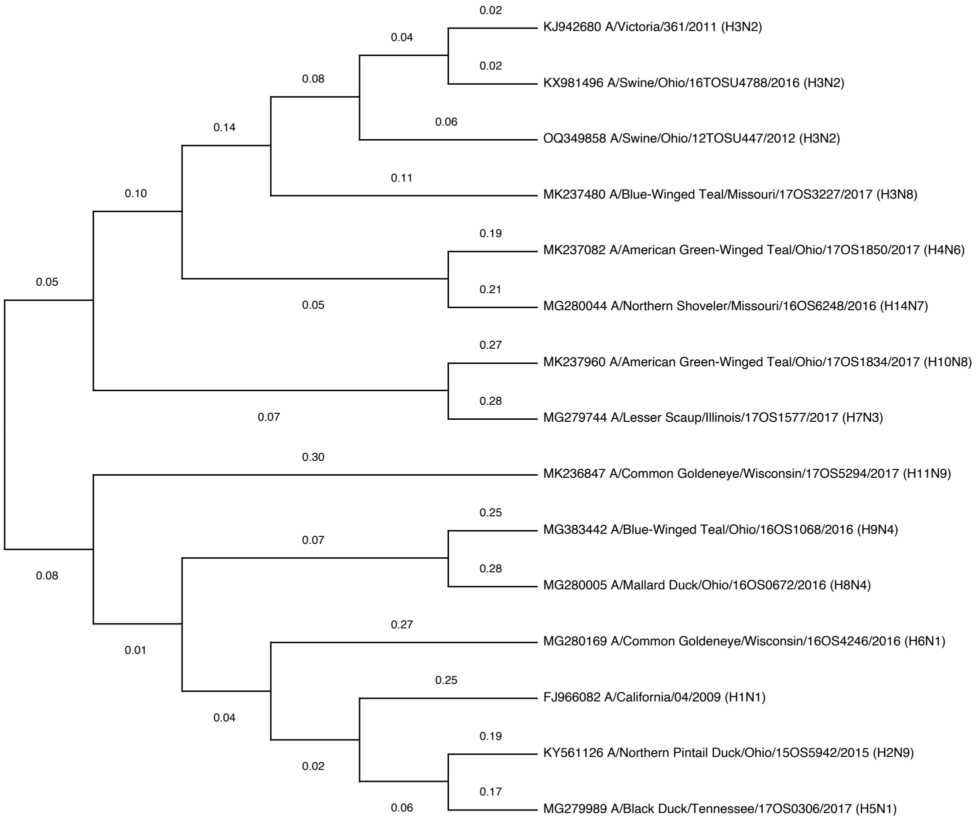

Supplementary Figure 1. Phylogenetic tree of avian-, swine-, and human-origin influenza viruses utilized in this study. Alignments and tree estimates for whole HA genomic segments were performed using Molecular Evolutionary Genetics Analysis (MEGA) software. Values represent the number of nucleotide substitutions normalized to the length of the sequences. The letter number combinations, such as MG279989, are the Genbank IDs for the HA gene segments of the individual viruses that were used to construct this phylogenetic table.

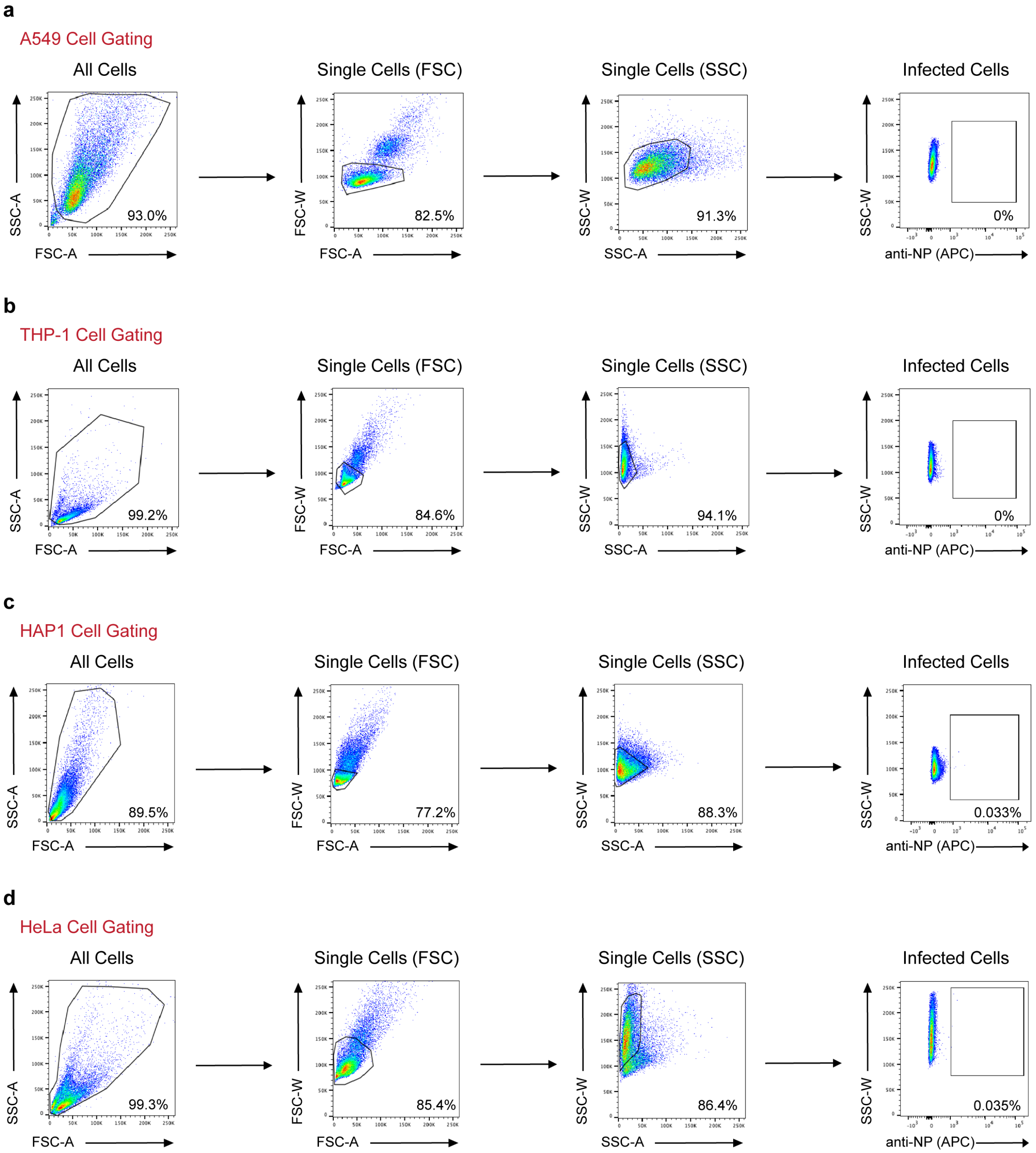
a
A549 Cell Gating
All Cells
Single Cells (FSC)
Single Cells (SSC)
Infected Cells
SSC-A
FSC-A
93.0%
FSC-W
FSC-A
82.5%
SSC-W
SSC-A
91.3%
SSC-W
anti-NP (APC)
0%
b
THP-1 Cell Gating
All Cells
Single Cells (FSC)
Single Cells (SSC)
Infected Cells
99.2%
84.6%
94.1%
0%
c
HAP1 Cell Gating
All Cells
Single Cells (FSC)
Single Cells (SSC)
Infected Cells
89.5%
77.2%
88.3%
0.033%
d
HeLa Cell Gating
All Cells
Single Cells (FSC)
Single Cells (SSC)
Infected Cells
99.3%
85.4%
86.4%
0.035%

Supplementary Figure 2. Gating strategy for determining percent infection. a-d Representative flow cytometry dot plots displaying the gating strategy utilized to analyze percent infection. All cell types were gated on non-infected control cells, the gates were then uniformly applied to all samples. First, all cells were gated on FSC-A versus SSC-A, the resulting population was used to then gate out single cells. Single cells were gated first on FSC-W versus FSC-A and that population was subsequently gated on SSC-W versus SSC-A. Finally the single cell population was gated for infected cells on SSC-W versus APC-A (the channel detecting the anti-influenza nucleoprotein with secondary antibody labeled with Alexafluor 647.). (**a**) A549 cells. (**b**) THP-1 cells. (**c**) HAP1 cells. (**d**) HeLa cells.

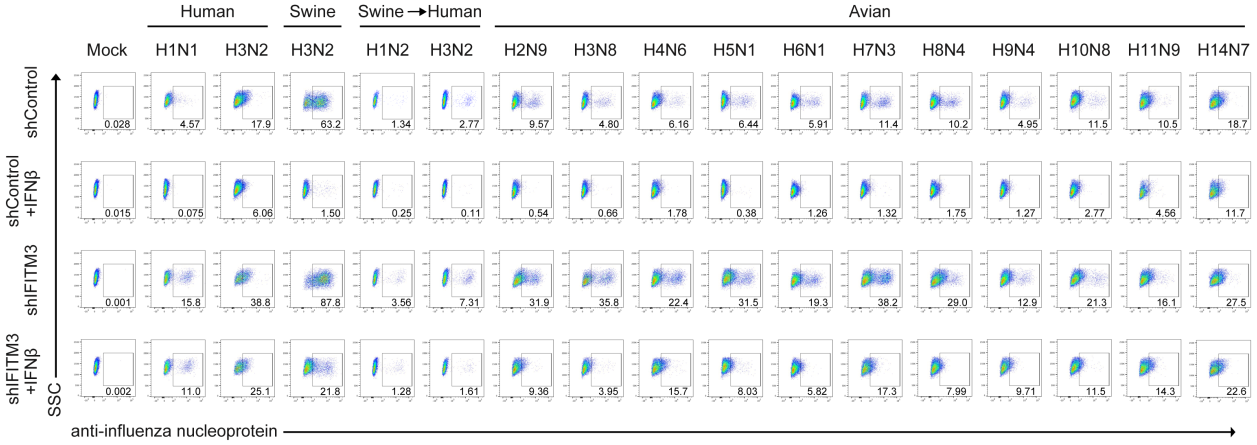

Supplementary Figure 3. Example raw flow cytometry data for determination of normalized infection of control (shControl) and IFITM3-knockdown (shIFITM3) A549 lung cells +/- IFNb treatment with each of the indicated virus strains as in main text Figure 1. SSC, side scatter; Anti-influenza nucleoprotein antibody was detected in the APC channel with secondary antibody labeled with Alexafluor 647.

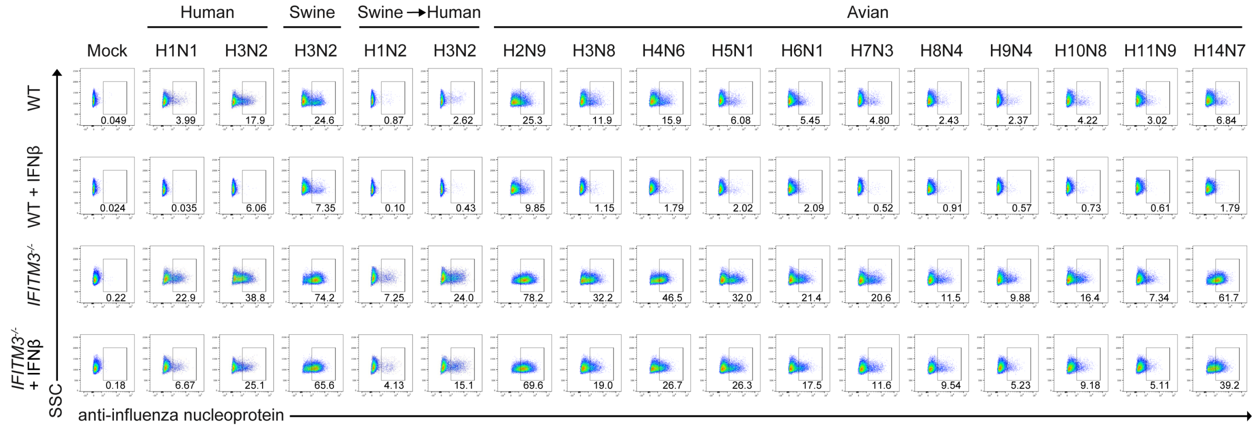

Supplementary Figure 4. Example raw flow cytometry data for determination of normalized infection of WT and *IFITM3$^{-/-}$* THP-1 macrophages +/- IFNb treatment with each of the indicated virus strains as in main text Figure 1. SSC, side scatter; Anti-influenza nucleoprotein antibody was detected in the APC channel with secondary antibody labeled with Alexafluor 647.

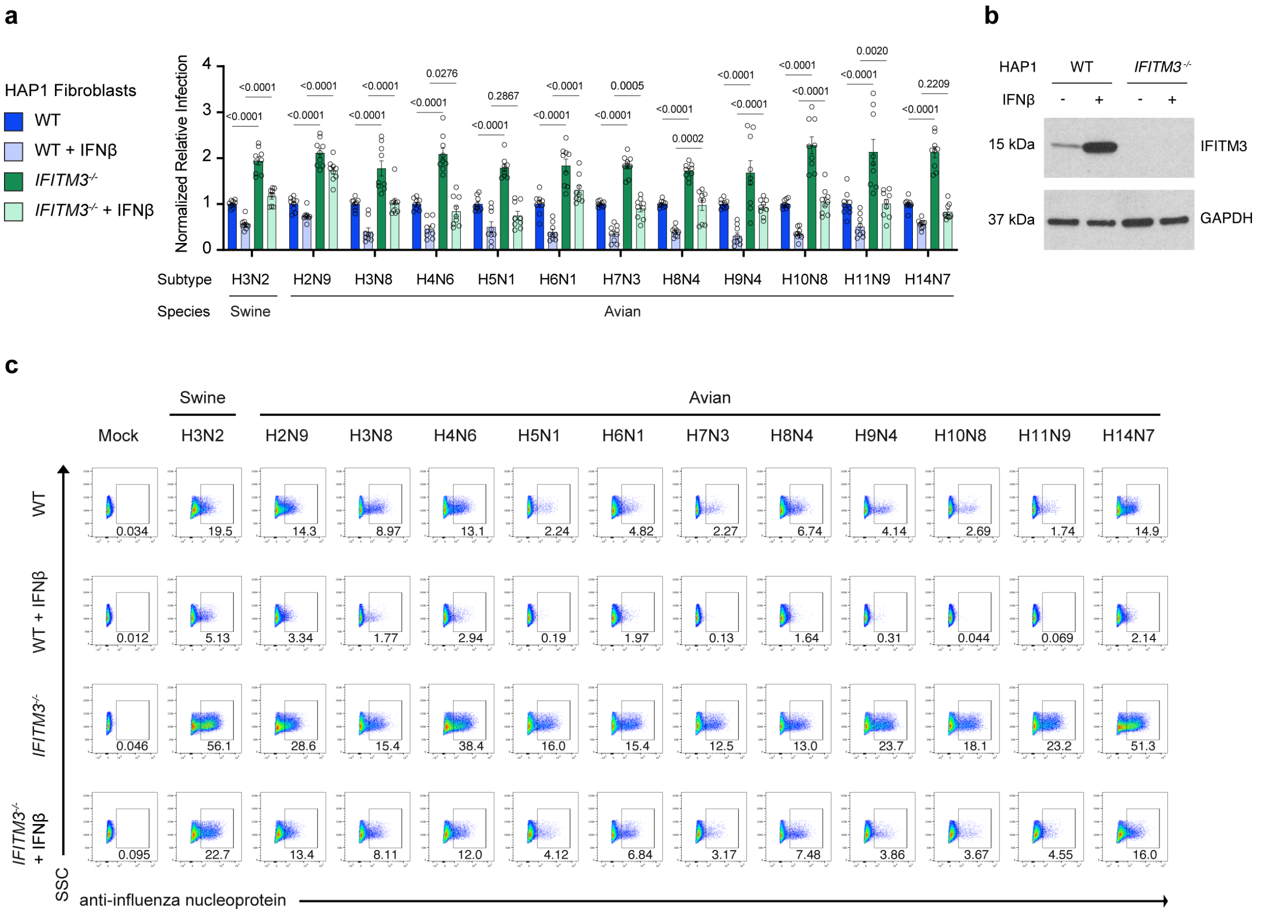

Supplementary Figure 5. IFITM3 limits animal-origin influenza virus infection of human fibroblast cells. HAP1 cells were treated +/- IFNβ for 18 hours, followed by infection with indicated viruses (MOI 1) for 24 hours. (**a**) Percent infection was determined by flow cytometry and normalized to results for WT cells without IFNβ pre-treatment. Error bars represent SEM. Exact p values are for the indicated comparisons and were determined by one-way ANOVA followed by Tukey's multiple comparisons test. Only statistical comparisons between WT versus *IFITM3*-/- are shown. Data are representative of 3 independent experiments each performed in triplicate (n=9). (**b**) Western blots of cell lysates at 18 hours +/- IFNβ treatment. (**c**) Example raw flow cytometry data for determination of normalized infection of WT and *IFITM3*-/- HAP1 cells +/- IFNβ treatment with each of the indicated virus strains as in (**a**). SSC, side scatter; Anti-influenza nucleoprotein antibody was detected in the APC channel with secondary antibody labeled with Alexafluor 647. Source data (**a, b**) are provided as a Source Data file. Gating strategy in **Supplementary Figure 2. c.**

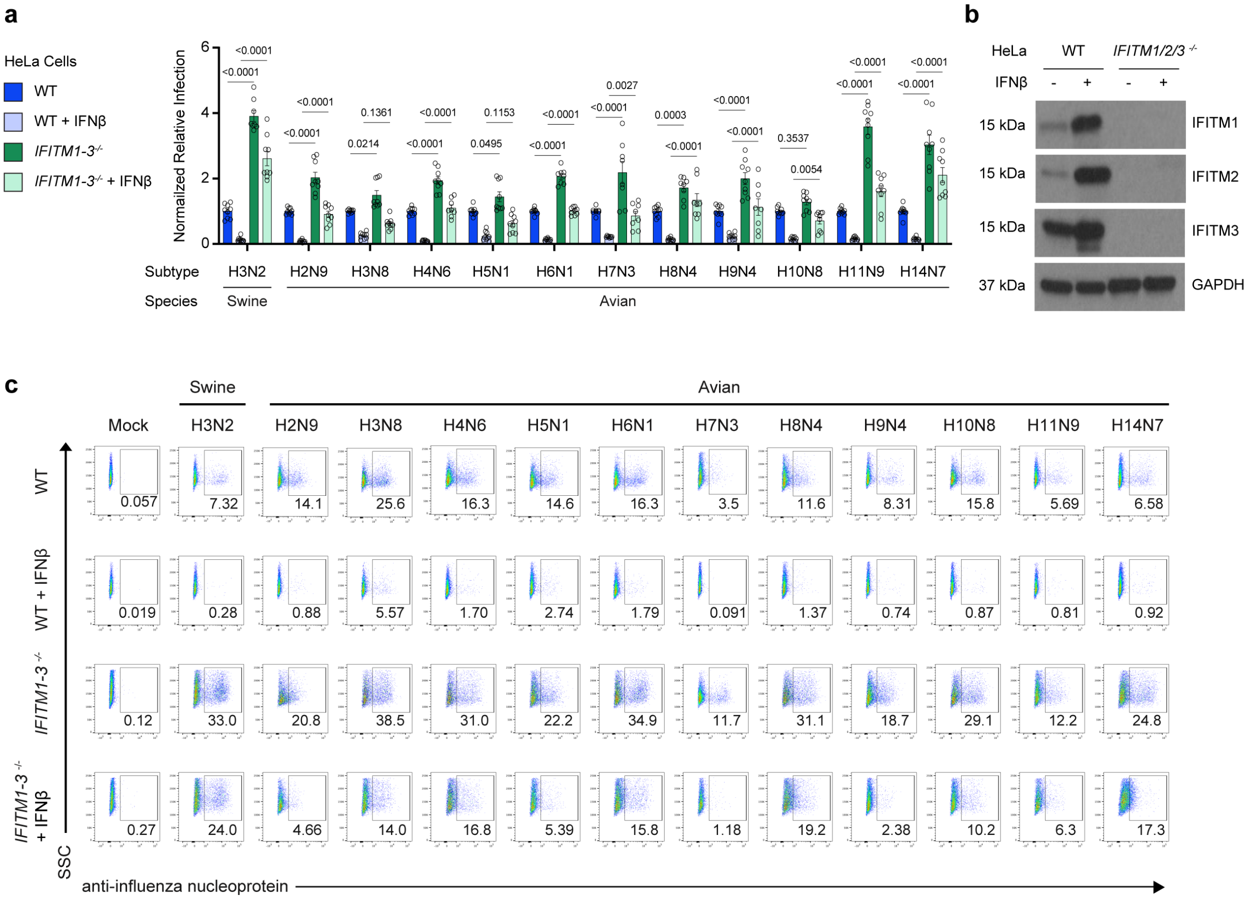

Supplementary Figure 6. IFITM3 limits animal-origin influenza virus infection of HeLa cells. HeLa cells were treated +/- IFNβ for 18 hours, followed by infection with indicated viruses (MOI 1) for 24 hours. (**a**) Percent infection was determined by flow cytometry and normalized to results for WT cells without IFNβ pre-treatment. Error bars represent SEM. P values shown are for the indicated comparisons and were determined by one-way ANOVA followed by Tukey's multiple comparisons test. Only statistical comparisons between WT versus *IFITM3*-/- are shown. Data are representative of 3 independent experiments each performed in triplicate (n=9). (**b**) Western blots of cell lysates at 18 hours +/- IFNβ treatment. (**c**) Example raw flow cytometry data for determination of normalized infection of WT and *IFITM1/2/3*-/- HeLa cells +/- IFNb treatment with each of the indicated virus strains as in (**a**). SSC, side scatter; Anti-influenza nucleoprotein antibody was detected in the APC channel with secondary antibody labeled with Alexafluor 647. All numbers above the graph represet exact p values. Source data (**a, b**) are provided as a Source Data file. Gating strategy in **Supplementary Figure 2. d.**

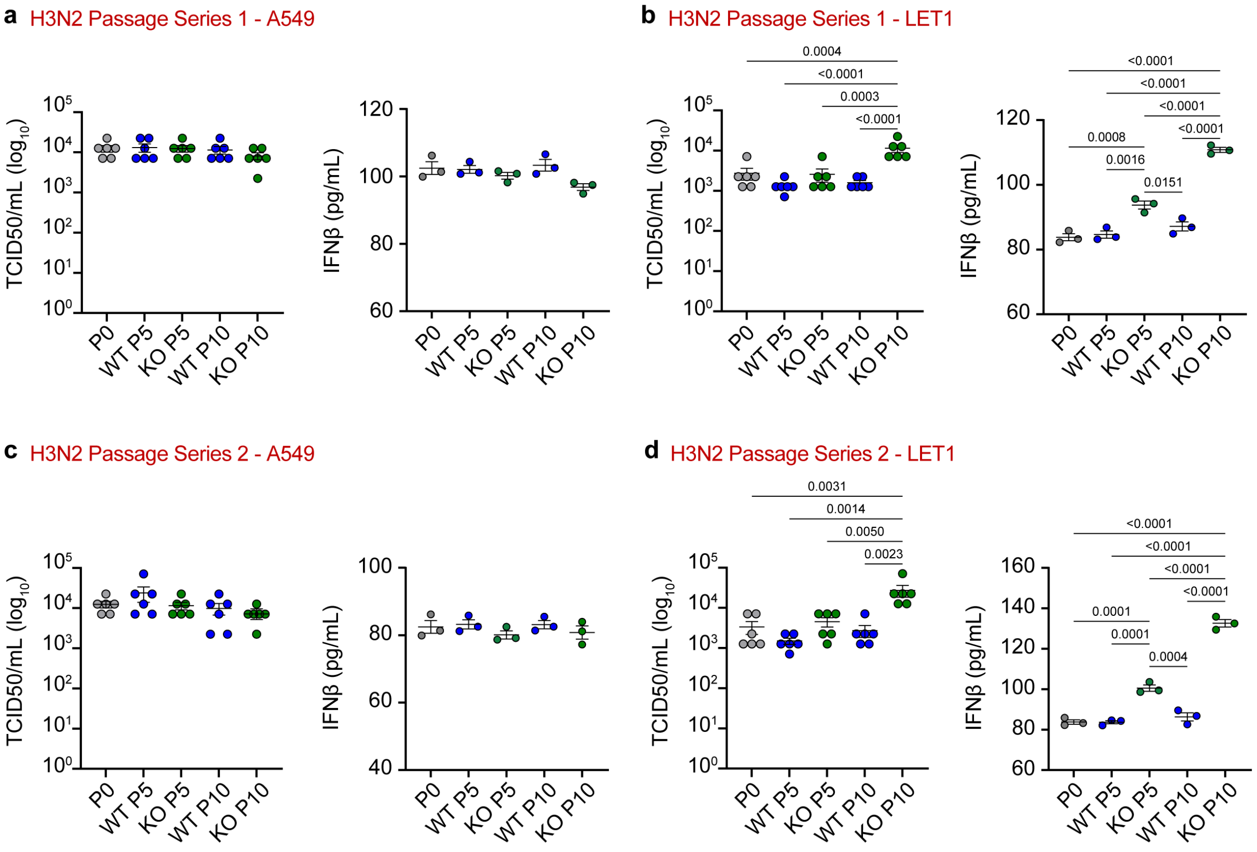

Supplementary Figure 7 . Increased viral replication and cytokine production of IFITM3 KO passaged A/Victoria/361/2011 (H3N2) virus in LET1 cells versus A549 cells. A549 or LET1 cells were infected with the indicated influenza viruses at an MOI of 1 and incubated in media containing TPCK-Trypsin for 48 hours to allow for multi-cycle replication. The supernatants were collected to determine viral titer and IFNβ levels were determined by ELISA. (**a,b**) H3N2 passaging series 1. (**c,d**) H3N2 passaging series 2. Titer data is representative of 2 independent experiments each performed in triplicate (n=6) and each dot in the ELISA data indicates an independent cell infection (n=3). Only comparisons between control and knockdown cells are shown for each virus. All error bars represent SEM. Comparisons were analyzed by ANOVA followed by Tukey's multiple comparisons test with numbers shown above the graph representing exact p values. Source data are provided as a Source Data file.

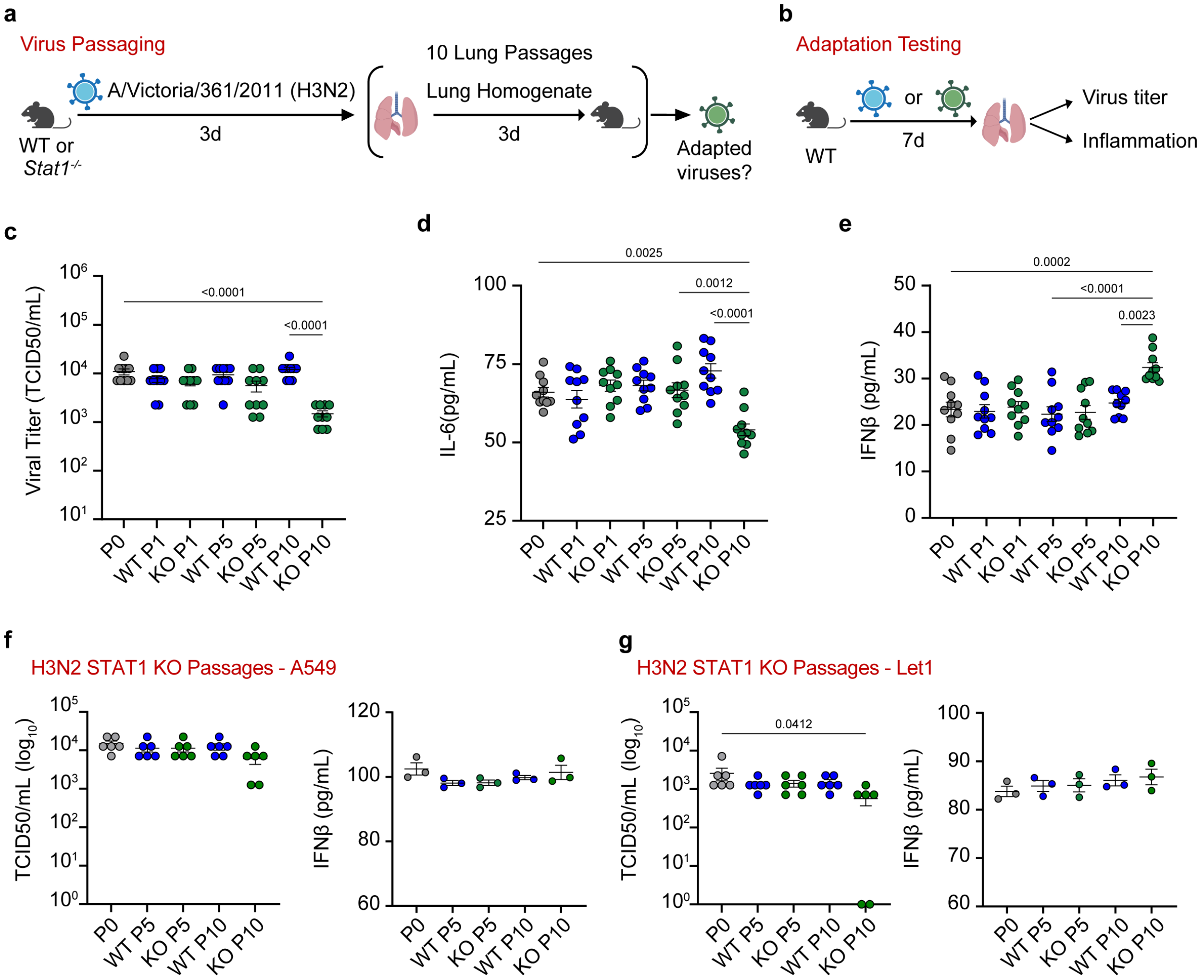
a
Virus Passaging
A/Victoria/361/2011 (H3N2)
3d
WT or
Stat1-/-
10 Lung Passages
Lung Homogenate
3d
Adapted
viruses?
b
Adaptation Testing
or
7d
WT
Virus titer
Inflammation
c
Viral Titer (TCID50/mL)
<0.0001
<0.0001
P0
WT P1
KO P1
WT P5
KO P5
WT P10
KO P10
d
IL-6(pg/mL)
0.0025
0.0012
<0.0001
e
IFNβ (pg/mL)
0.0002
<0.0001
0.0023
f
H3N2 STAT1 KO Passages - A549
TCID50/mL (log10)
IFNβ (pg/mL)
P0
WT P5
KO P5
WT P10
KO P10
g
H3N2 STAT1 KO Passages - Let1
TCID50/mL (log10)
0.0412
IFNβ (pg/mL)

Supplementary Figure 8. Influenza virus becomes attenuated when passaged in the absence of STAT1. (**a**) Schematic of mouse passaging experiments. Initial intranasal infections were performed with 1,000 $TCID_{50}$ of A/Victoria/361/2011 (H3N2). (**b**) Schematic of WT mouse challenged with 1,000 $TCID_{50}$ of the parental or passaged viruses. (**c,d**) Groups of WT mice were challenged with equal doses of virus passaged 1, 5, or 10 times through WT or *Stat1*$^{-/-}$ mice and compared to the parent virus (passage 0). (**c**) Viral titers from lung homogenates collected at day 7 post infection. Error bars represent SEM. Comparisons were analyzed by ANOVA followed by Tukey's multiple comparisons test (**d,e**) ELISA quantification of IL-6 (**d**) levels in lung homogenates of WT and IFITM3 KO mice at day 7 post infection**.** All error bars represent SEM. Comparisons were analyzed by one-way ANOVA followed by Tukey's multiple comparisons test. **c-e** represents 2 independent experiments (n=10). (**f,g**) A549 or LET1 cells were infected with the indicated influenza viruses at an MOI of 1 and incubated in media containing TPCK-Trypsin for 48 hours to allow for multi-cycle replication. The supernatants were collected to determine viral titer and IFNβ levels were determined by ELISA. Titer data is representative of 2 independent experiments each performed in triplicate (n=6) and each dot in the ELISA data indicates an independent cell infection (n=3). Only comparisons between control and knockdown cells are shown for each virus. All error bars represent SEM. Comparisons were analyzed by one-way ANOVA followed by Tukey's multiple comparisons test. All numbers shown above the graphs represent exact p values. Source data (**c-g**) are provided as a Source Data file. (**a, b**) Created in BioRender. Denz, P. (2024) BioRender.com/r93l474

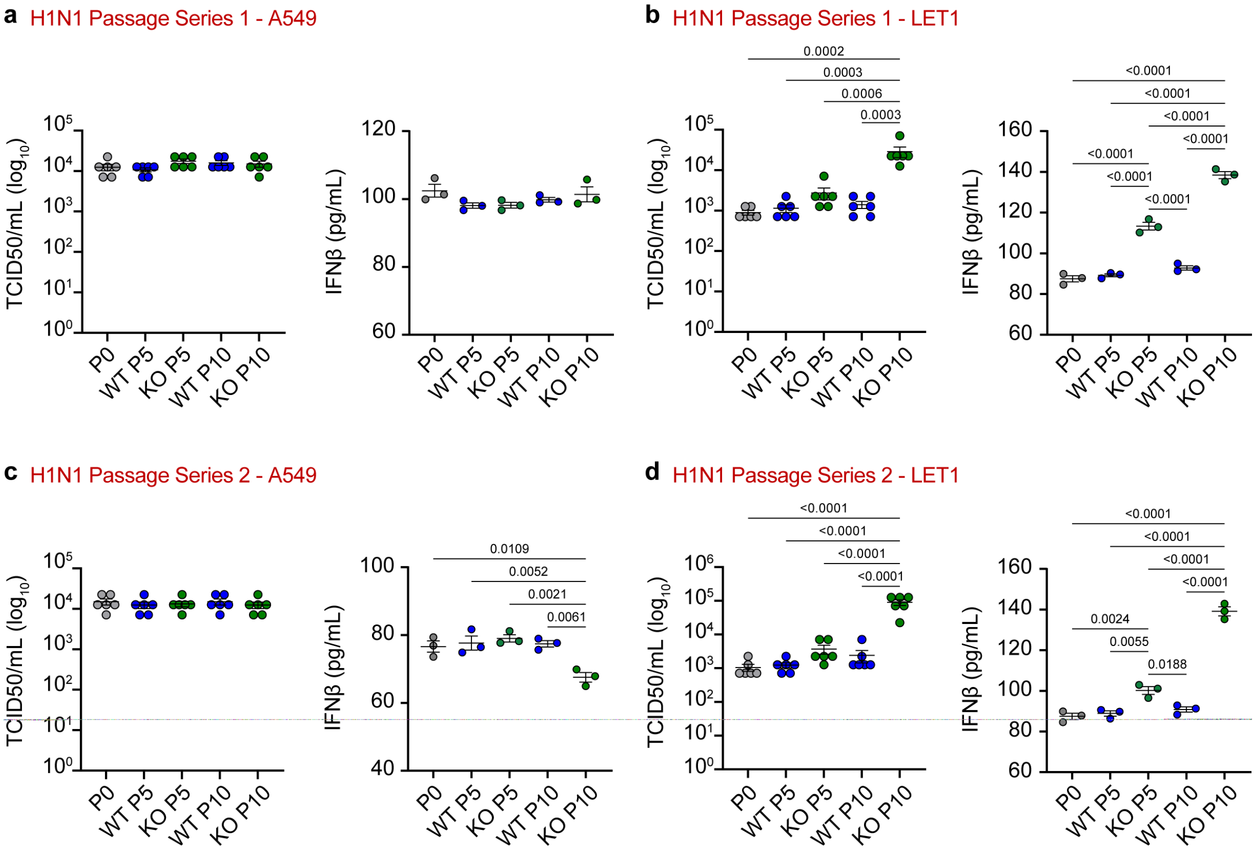

Supplementary Fig 9. Increased viral replication and cytokine production of IFITM3 KO passaged A/California/04/2009 (H1N1) virus in LET1 cells versus A549 cells. A549 or LET1 cells were infected with the indicated influenza viruses at an MOI of 1 and incubated in media containing TPCK-Trypsin for 48 hours to allow for multi-cycle replication. The supernatants were collected to determine viral titer and IFNβ levels were determined by ELISA. (**a,b**) H1N1 passaging series 1. (**c,d**) H1N1 passaging series 2. Titer data is representative of 2 independent experiments each performed in triplicate (n=6) and each dot in the ELISA data indicates an independent cell infection (n=3). Only comparisons between control and knockdown cells are shown for each virus. All error bars represent SEM. Comparisons were analyzed by one-way ANOVA followed by Tukey's multiple comparisons test. All numbers shown above the graphs represent exact p values. Source data are provided as a Source Data file.

Influenza Virus Strain	Treated TRBC (α-2,6) Titer	TRBC (α-2,6/α-2,3) Titer	HRBC (α-2,3) Titer	Receptor Preference
A/California/04/2009 (H1N1), Human	512	512	<2	α-2,6
A/Victoria/361/2011 (H3N2), Human	2048	4069	<2	α-2,6
A/Swine/Ohio/18TOSU4536/2018 (H1N2)	256	512	8	α-2,6
A/Swine/Ohio/16TOSU4788/2016 (H3N2)	128	128	<2	α-2,6
A/Swine/Ohio/12TOSU0447/2012 (H3N2)	4069	1024	8	α-2,6
A/Northern Pintail Duck/Ohio/15OS5942/2015 (H2N9)	<2	64	32	α-2,3
A/Blue-Winged Teal/Missouri/17OS3227/2017 (H3N8)	<2	512	32	α-2,3
A/American Green-Winged Teal/Ohio/17OS1850/2017 (H4N6)	<2	1024	1024	α-2,3
A/Black Duck/Tennessee/17OS0306/2017 (H5N1)	<2	32	32	α-2,3
A/Common Goldeneye/Wisconsin/16OS4246/2016 (H6N1)	32	32	32	Both
A/Lesser Scaup/Illinois/17OS1577/2017 (H7N3)	4	128	128	α-2,3
A/Mallard Duck/Ohio/16OS0672/2016 (H8N4)	<2	<2	<2	ND
A/Blue-Winged Teal/Ohio/16OS1068/2016 (H9N4)	64	64	32	Both
A/American Green-Winged Teal/Ohio/17OS1834/2017 (H10N8)	8	8	8	Both
A/Common Goldeneye/Wisconsin/17OS5294/2017 (H11N9)	2048	2048	2048	Both
A/Northern Shoveler/Missouri/16OS6248/2016 (H14N7)	<2	32	32	α-2,3

Supplementary Table 1. Virus strains utilized in this study and their sialic acid receptor binding preferences. Data shown are hemagglutination titers used to determine virus sialic acid receptor binding preferences. TRBC, turkey red blood cell; Treated TRBC indicates TRBC incubated with a neuraminidase enzyme to remove a-2,3 sialic acid linkages; HRBC, horse red blood cell; ND, not determinable.

Uncropped scans of Western Blots:

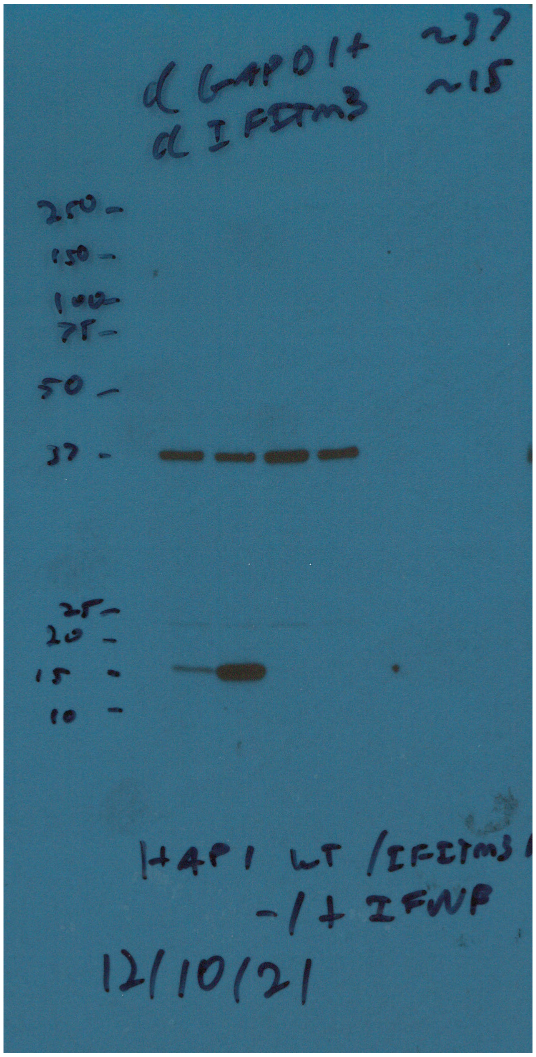

Supplementary Figure 4b. HAP1 cell westerns. anti-GAPDH and anti-IFITM3 antibodies used to probe whole cell protein lysates (30 second exposure). Protein ladder 10-250 kDa, lanes (left to right) HAP1 WT, HAP1 WT + IFNβ, HAP1 *IFITM3*$^{-/-}$, HAP1 *IFITM3*$^{-/-}$ +IFNβ.

a.

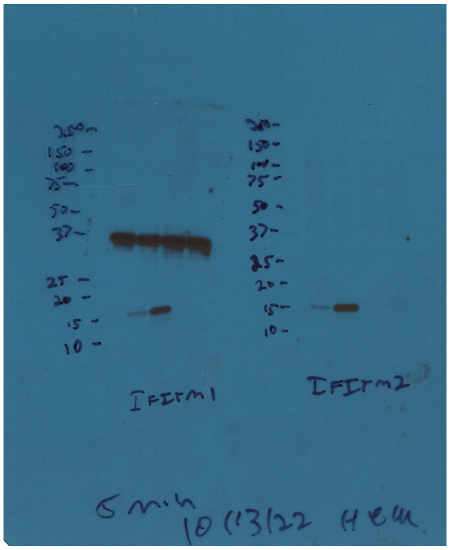

b.

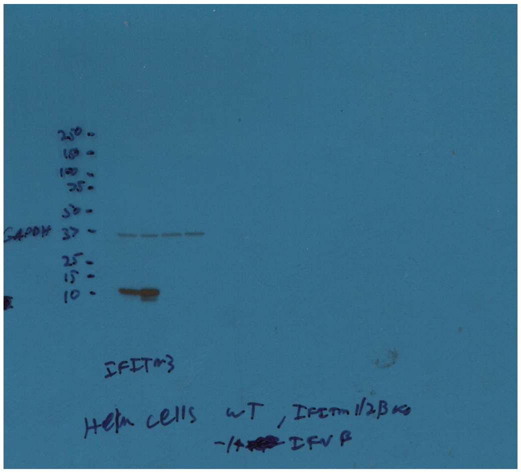

c.

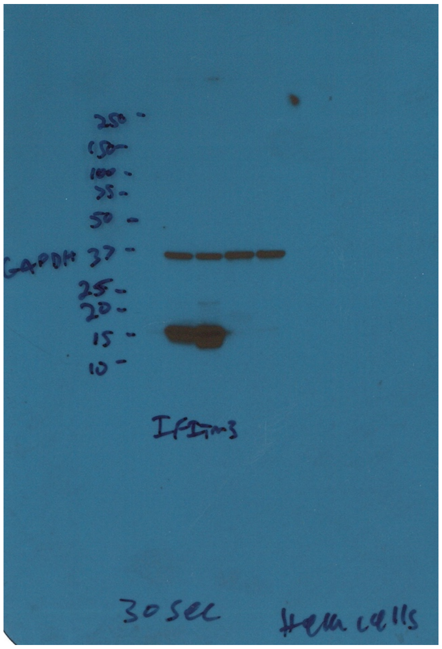

Supplementary Figure 5b HeLa cell westerns. anti-IFITM1, anti-IFITM2, anti-IFITM3, and anti-GAPDH antibodies used to probe whole cell protein lysates (30 second exposure and 5 minute exposure). Protein ladder 10-250 kDa, lanes (left to right) HeLa WT, HeLa WT + IFNβ, HeLa *IFITM1-3*$^{-/-}$, HeLa *IFITM1-3*$^{-/-}$ +IFNβ. **a.** anti-GAPDH, anti-IFITM1, anti-IFITM2 (5 minute exposure). **b.** anti-GAPDH and anti-IFITM3 (instant exposure). **c.** anti-GAPDH and anti-IFITM3 (30 second exposure).
