# Supplementary material for: Innate immune control of influenza virus interspecies adaptation via IFITM3
Source: Nat Commun. 2024 Oct 30;15:9375. doi: 10.1038/s41467-024-53792-3 (PMC11525587; doi:10.1038/s41467-024-53792-3)
Supplement: Supplementary file 2 — Description of Additional Supplementary Files [file 41467_2024_53792_MOESM2_ESM.docx]

**Description of Additional Supplementary Files**

**Supplementary Data 1:** Full list of variants above 1% frequency in the A/Victoria/361/2011 (H3N2) parent virus stock. This table also lists the frequency of the variants in passages 5 and 10 from WT, *Ifitm3^-/-^*, and *Stat1^-/-^* mice.

**Supplementary Data 2:** Full list of variants above 1% frequency in passages 5 and 10 from WT and *Ifitm3^-/-^* mice compared to the A/Victoria/361/2011 (H3N2) parent virus stock.

**Supplementary Data 3:** Full list of variants above 1% frequency in passages 5 and 10 from WT and *Stat1^-/-^* mice compared to the A/Victoria/361/2011 (H3N2) parent virus stock.

**Supplementary Data 4:** Full list of variants above 1% frequency in the A/California/04/2009 (H1N1) parent virus stock compared to the consensus sequence for this virus. The table also lists the frequency of the variants present in passages 5 and 10 from WT and *Ifitm3^-/-^* mice.

**Supplementary Data 5:** Full list of variants above 1% frequency in passages 5 and 10 from WT and *Ifitm3^-/-^* mice compared to the A/California/04/2009 (H1N1) parent virus stock.
