# Supplementary material for: Innate immune control of influenza virus interspecies adaptation via IFITM3
Source: Nat Commun. 2024 Oct 30;15:9375. doi: 10.1038/s41467-024-53792-3 (PMC11525587; doi:10.1038/s41467-024-53792-3)
Supplement: Supplementary file 8 — Reporting Summary [file 41467_2024_53792_MOESM8_ESM.pdf]

nature portfolio

Corresponding author(s): Jacob S. Yount

Last updated by author(s): Oct 17, 2024

Reporting Summary

Nature Portfolio wishes to improve the reproducibility of the work that we publish. This form provides structure for consistency and transparency in reporting. For further information on Nature Portfolio policies, see our Editorial Policies and the Editorial Policy Checklist.

Statistics

For all statistical analyses, confirm that the following items are present in the figure legend, table legend, main text, or Methods section.

n/a	Confirmed	
[ ]	[x]	The exact sample size (n) for each experimental group/condition, given as a discrete number and unit of measurement
[ ]	[x]	A statement on whether measurements were taken from distinct samples or whether the same sample was measured repeatedly
[ ]	[x]	The statistical test(s) used AND whether they are one- or two-sided<br>*Only common tests should be described solely by name; describe more complex techniques in the Methods section.*
[x]	[ ]	A description of all covariates tested
[ ]	[x]	A description of any assumptions or corrections, such as tests of normality and adjustment for multiple comparisons
[ ]	[x]	A full description of the statistical parameters including central tendency (e.g. means) or other basic estimates (e.g. regression coefficient) AND variation (e.g. standard deviation) or associated estimates of uncertainty (e.g. confidence intervals)
[ ]	[x]	For null hypothesis testing, the test statistic (e.g. F, t, r) with confidence intervals, effect sizes, degrees of freedom and P value noted<br>*Give P values as exact values whenever suitable.*
[x]	[ ]	For Bayesian analysis, information on the choice of priors and Markov chain Monte Carlo settings
[ ]	[x]	For hierarchical and complex designs, identification of the appropriate level for tests and full reporting of outcomes
[x]	[ ]	Estimates of effect sizes (e.g. Cohen's d, Pearson's r), indicating how they were calculated

Our web collection on statistics for biologists contains articles on many of the points above.

Software and code

Policy information about availability of computer code

Data collection	BD FACSDiva™ Software v. 6.1.3, SoftMax Pro Software v7
Data analysis	Graphpad Prism v9, Molecular Evolutionary Genetics Analysis (MEGA) software v11.0.13, FlowJo Software (version 10.8.1) ,CLC Genomics Workbench (version 22.0.1)

For manuscripts utilizing custom algorithms or software that are central to the research but not yet described in published literature, software must be made available to editors and reviewers. We strongly encourage code deposition in a community repository (e.g. GitHub). See the Nature Portfolio guidelines for submitting code & software for further information.

Data

Policy information about availability of data

All manuscripts must include a data availability statement. This statement should provide the following information, where applicable:

- Accession codes, unique identifiers, or web links for publicly available datasets
- A description of any restrictions on data availability
- For clinical datasets or third party data, please ensure that the statement adheres to our policy

The raw data that support the findings of this study are available in the Source Data document with sequencing data available at GenBank under the accession numbers:PQ384645 - PQ384820. https://www.ncbi.nlm.nih.gov/nuccore/?term=PQ384645:PQ384820[accn]

Research involving human participants, their data, or biological material

Policy information about studies with human participants or human data. See also policy information about sex, gender (identity/presentation), and sexual orientation and race, ethnicity and racism.

Reporting on sex and gender	No human participants in this study
Reporting on race, ethnicity, or other socially relevant groupings	No human participants in this study
Population characteristics	No human participants in this study
Recruitment	No human participants in this study
Ethics oversight	No human participants in this study

Note that full information on the approval of the study protocol must also be provided in the manuscript.

Field-specific reporting

Please select the one below that is the best fit for your research. If you are not sure, read the appropriate sections before making your selection.

[x] Life sciences [ ] Behavioural & social sciences [ ] Ecological, evolutionary & environmental sciences

For a reference copy of the document with all sections, see nature.com/documents/nr-reporting-summary-flat.pdf

Life sciences study design

All studies must disclose on these points even when the disclosure is negative.

Sample size	No statistical method was used to predetermine sample size. For in vivo experiments, we based the number of mice on preliminary studies that determined experimental variation in survival and weight loss following infection. For in vitro experiments, assays were performed using the indicated sample sizes to ensure reproducibility.
Data exclusions	No data were excluded from analysis.
Replication	The reported findings have been confirmed in large groups of mice/experimental group in multiple (>2) experiments. Mouse passaging studies utilized two influenza strains to minimize strain-specific effects. In vitro infections were recapitulated in four human cell lines further confirming the reproducibility of the results.
Randomization	Prior to infection, we ensured that the mean weight and age were comparable among the various groups of mice. Each group was randomly assigned which virus they were challenged with. No randomization was used for the in vitro experiments, as it is not applicable to the study design.
Blinding	Blinding was not performed for this study.

Reporting for specific materials, systems and methods

We require information from authors about some types of materials, experimental systems and methods used in many studies. Here, indicate whether each material, system or method listed is relevant to your study. If you are not sure if a list item applies to your research, read the appropriate section before selecting a response.

Materials & experimental systems

n/a	Involved in the study	
[ ]	[x]	Antibodies
[ ]	[x]	Eukaryotic cell lines
[x]	[ ]	Palaeontology and archaeology
[ ]	[x]	Animals and other organisms
[x]	[ ]	Clinical data
[x]	[ ]	Dual use research of concern
[x]	[ ]	Plants

Methods

n/a	Involved in the study	
[x]	[ ]	ChIP-seq
[ ]	[x]	Flow cytometry
[x]	[ ]	MRI-based neuroimaging

Antibodies

Antibodies used	IFITM1 (Cell Signaling Technology, #13126), IFITM2 (Cell Signaling Technology, #13530), IFITM3 (ProteinTech, 11714-1-AP), GAPDH

	(Thermo Scientific, ZG003), Influenza A Virus Nucleoprotein (Abcam, ab20343), Goat anti-Mouse IgG (H+L) Highly Cross-Adsorbed Secondary Antibody, Alexa Fluor 647 (Thermo Scientific, Catalog # A-21236). These antibodies were used at dilutions of 1:1000 as determined by preliminary experiments optimizing the protocol for flow cytometry and western blotting. Statements of the antibody dilutions have been added to the appropriate sections of the Methods portion of the manuscript.
Validation	All antibodies used are commercially available and validation has been performed by the manufacturer. Corresponding certificates of analysis are available at the manufacturer's website.

Eukaryotic cell lines

Policy information about cell lines and Sex and Gender in Research

Cell line source(s)	A549 knockdown cells were generated by lentiviral shRNA-mediated targeting, THP-1 IFITM3 knockout cells were generated via CRISPR-Cas9 targeting (provided by Dr. Anasuya Sarkar of the Ohio State University). HeLa IFITM1/2/3 knockout and HAP1 IFITM3 knockout were purchased from ATCC (CRL-3452) and Horizon Discovery Biosciences (HZGHC004186c010), respectively. MDCK and LET1 cells were obtained from BEI Resources (NR-2628 and NR-42941 respectively).
Authentication	Original cell stocks maintained in liquid N2 storage and each thawed aliquot discarded after 20 cell passages. Knockdown and Knockout cells were authenticated via western blot, see figure 1 and extended figures 4,5.
Mycoplasma contamination	No contamination present, confirmed negative of mycoplasma using the Lonza Mycoalert detection kit. All cell lines were maintained for <20 passages to minimize mycoplasma contamination.
Commonly misidentified lines (See ICLAC register)	None used

Animals and other research organisms

Policy information about studies involving animals; ARRIVE guidelines recommended for reporting animal research, and Sex and Gender in Research

Laboratory animals	Male and female mice between 6 and 10 weeks of age were used in our experiments. All mice used in this study were of the C57BL/6J background. Ifitm3-/- mice with a 53 base pair deletion in exon 1 of the Ifitm3 gene were described previously23. WT mice for comparison to Ifitm3-/- mice were obtained from Charles River Laboratories. Stat1-/- mice (Strain #: 012606) and complementary WT C57BL/6J mice (strain #007914) were obtained from The Jackson Laboratory. Mice were housed in the Ohio State University's Biomedical Research Tower vivarium, which is maintained at 68–76 degrees F, with a 12:12 light dark cycle, and humidity between 30–70%. Autoclaved individually ventilated cages (Allentown) were used for housing. Mice were fed irradiated natural ingredient chow ad libitum (Evnigo Teklad Diet 7912). Reverse osmosis purified water was provided through an automated rack water system. Cages included 1⁄4 inch of corn cob bedding (Bed-o-Cobs, The Andersons) with cotton square nesting material.
Wild animals	None used
Reporting on sex	Both male and female mice were used in our experiments and we did not collect any sex-specific data.
Field-collected samples	Swine and avian viruses were collected by Andrew Bowman of the Ohio State University and were also propagated in embryonated chicken eggs (Charles River Laboratories)
Ethics oversight	All procedures were approved by the Ohio State University IACUC and were performed in accordance with guidelines for the ethical use of animals under protocol number 2016A00000051-R2

Note that full information on the approval of the study protocol must also be provided in the manuscript.

Flow Cytometry

Plots

Confirm that:

- [x] The axis labels state the marker and fluorochrome used (e.g. CD4-FITC).
- [x] The axis scales are clearly visible. Include numbers along axes only for bottom left plot of group (a 'group' is an analysis of identical markers).
- [x] All plots are contour plots with outliers or pseudocolor plots.
- [x] A numerical value for number of cells or percentage (with statistics) is provided.

Methodology

Sample preparation	Cells were collected 24 hours post infection and fixed in 4% paraformaldehyde for 10 minutes at room temperature, permeabilized with 0.1% Triton X-100 in PBS, stained with an antibody against Influenza A Virus Nucleoprotein (Abcam, ab20343) conjugated with Goat anti-Mouse IgG (H+L) Highly Cross-Adsorbed Secondary Antibody, Alexa Fluor™ 647 (Thermo Scientific, Catalog # A-21236) resuspended in 2% Fetal Bovine Serum in Phosphate Buffered Saline.

Instrument	BD FACS CANTO II
Software	BD FACSDiva™ Software v. 6.1.3 and FlowJo Software (version 10.8.1) were used to collect and analyze all samples.
Cell population abundance	2x10^4 cells per sample, see supplementary figures 2,3,4, 5 and 6
Gating strategy	Gating strategy is presented in supplementary figure 2. Preliminary gating was performed as FSC-A/SSC-A, singlets were then gated on within that population using FSC-W/FSC-A. To ensure only single cells are within our population we also gated SSC-W/SSC-A. Matched uninfected controls were used to create the infected cells gate on SSC-W/APC-A, therefore infected cells are indicated as APC+ cells.

☒ Tick this box to confirm that a figure exemplifying the gating strategy is provided in the Supplementary Information.
